# Differential social evaluation of pregnant teens, teen mothers and teen fathers by university students

**DOI:** 10.1080/02673843.2014.963630

**Published:** 2014-10-01

**Authors:** Keri Weed, Jody S. Nicholson

**Affiliations:** ^a^ Department of Psychology, University of South Carolina Aiken, Aiken, SC, USA; ^b^ Department of Psychology, University of North Florida, Jacksonville, FL, USA

**Keywords:** teen pregnancy and parenting, teen fathers, social evaluation, attitudes, stereotypes

## Abstract

Youth may be particularly attuned to social evaluation during the teen years with implications for physical and mental health. Negative attitudes and stereotypes constitute an important type of social evaluative threat. Pregnant and parenting teens not only encounter challenges associated with their early transition to parenthood, but also are confronted with unfavourable attitudes of others. A university sample of 255 men and women responded to surveys targeting their feelings and beliefs about pregnant teens, teen mothers and teen fathers. Teen mothers were generally perceived more positively than pregnant teens who were perceived more positively compared to teen fathers. Social evaluations were generally unrelated to respondents' sex or race, but respondents who had contact with a friend or family member who had experienced a teen pregnancy were selectively more positive, as were freshmen compared to seniors. Risks attributed to early childbearing may be exacerbated by negative social evaluations.

The role of stereotypes in the lives of pregnant and parenting teens has received only cursory attention despite the potential of negative social evaluations to exacerbate challenges associated with an early transition to parenthood. Qualitative and quantitative studies have confirmed that many pregnant and parenting teens feel stereotyped and stigmatised (Kelly, 1996, 1997; Wiemann, Rickert, Berenson, & Volk, 2005; Yardley, 2008), and that these feelings may interfere with their personal well-being and the well-being of their children (Atuyambe, Mirembe, Annika, Kirumira, & Faxelid, 2009; Eisenberger, Lieberman, & Williams, 2003; Fulford & Ford-Gilboe, 2004; Hueston, Geesey, & Diaz, 2008; Masten et al., 2009; SmithBattle, 2013; Somerville, 2013). However, few studies have systematically investigated how pregnant and parenting teens are perceived by others. Furthermore, existing questionnaires focus on perceptions of teen mothers, and ignore perceptions of pregnant teens and teen fathers (Eshbaugh, 2011; Johansson & Hammarén, 2014; Kim, Burke, Sloan, & Barnett, 2013). The three goals of the current study were (1) to assess the social evaluations of pregnant and parenting teens by university students, (2) to compare the magnitude of negative evaluations of pregnant teens, teen mothers and teen fathers, and (3) to investigate individual differences among university students in social evaluations of pregnant and parenting teens.

## Social evaluations

Attitudes are evaluative reactions that are comprised of cognitive, affective and behavioural components (Millar & Tesser, 1992). The cognitive component may manifest as a stereotype or overgeneralised belief about the attributes of someone who belongs to a member of a group (e.g. the belief that pregnant and parenting teens are irresponsible). The affective component of attitudes reflects feelings towards members of the group (e.g. positive or negative emotions felt towards pregnant or parenting teens). Finally, the behavioural component includes an intention to act in specific ways in relation to the group member (e.g. to provide or withhold support). Collectively, these three components of attitudes directed towards members of an identified social group constitute social evaluations.

Cognitive, affective and behavioural aspects of attitudes may not always be congruent (Amodio & Devine, 2006; Devine, 1989; Esses & Dovidio, 2002; Millar & Tesser, 1992; Ramasubramanian, 2010). For example, positive feelings for cigarettes or candy are often accompanied by beliefs that consumption of these products entails health risks. Alternatively, negative feelings about an upcoming dental visit may exist concurrently with beliefs about the positive consequences associated with this action. Therefore, understanding social evaluations involves consideration of both affective and cognitive components and how they jointly impact behavioural decisions. Reducing stigma towards pregnant and parenting teens may require targeting not just the cognitive component of attitudes, but also the affective component (Millar & Tesser, 1992; Ramasubramanian, 2010). Furthermore, direct experience with a member of an out-group, termed the contact hypothesis, might differentially impact these cognitive and affective components of social evaluation (Jackson, 1993).

## Vulnerability to negative social evaluation

Stigma towards pregnant and parenting teens may constitute an ongoing social evaluative threat that directly impacts their developing self-concept and identity (Eisenberger et al., 2003; Fulford & Ford-Gilboe, 2004; Masten et al., 2009; Somerville, 2013). Blakemore and colleagues have focused their research on the neurobiological basis of how teens process social evaluation. Through structural and functional imagining, these researchers have shed light on the social cognition of the teenage brain, sometimes referred to as the *social brain* (Blakemore, 2008; Burnett, Sebastian, Kadosh, & Blakemore, 2011). The rise in social sensitivity during the teen years parallels development of the socio-affective circuitry of the brain, including the amygdala, striatum and medial prefrontal cortex. Implications of these developmental processes include increased awareness of the perspectives of others accompanied by increased vulnerability to negative social evaluations (Sebastian, Burnett, & Blakemore, 2008).

Consideration of the social brain during the teen years has provided clues to the reactions of youth to negative social evaluation. For teens, experimentally induced rejection, exclusion, and even just the threat of negative evaluation produced heightened self-conscious emotions, triggered stress reactions and led to negative mood and heightened anxiety (Sebastian, Viding, Williams, & Blakemore, 2010; Somerville, 2013). A meta-analysis of more than 200 research studies led to the conclusion that social-evaluative threat with uncontrollable outcomes elicited the largest increases in cortisol response accompanied by a longer delay to return to baseline (Dickerson & Kemeny, 2004). The authors suggested that continued exposure to social-evaluative threats may have long-term health implications due to cumulative stress. Other neuroimaging research has revealed that the pain of social exclusion is experienced similarly to physical pain, and that some teens may be less able to regulate their distress associated with social exclusion than adults (Masten et al., 2009). Teen parents who experience stigma from their pregnancy and off-timed parenting may be at heightened risk for health problems associated with social evaluative threat.

## Social evaluation of teen parents

Stigmatisation towards young parents is prevalent. In a study of low-income teen mothers living in south Texas, two out of five mothers reported feeling stigmatised (Wiemann et al., 2005). A study of Canadian teen mothers found an even higher prevalence of mothers experiencing stigma related to their youthful pregnancy (83%; Fulford & Ford-Gilboe, 2004). The stigma was more likely to come from strangers or health-care providers rather than from friends or family, although other studies have found teen mothers to report stigmatisation from people close to them (Kelly, 1996, 1997; Wiemann et al., 2005). Stigma was typically expressed non-verbally through facial expressions, but many of the study participants also reported receiving inappropriate verbal comments and rude treatment (Fulford & Ford-Gilboe, 2004).

Teen mothers have reported they are judged negatively for a variety of reasons, including their youth, not being fit to be a mother, immorality of being an unwed mother, presumption of substance use or criminal behaviour, irresponsible behaviour and dependence on the welfare system; they may be stereotyped as being irresponsible, ignorant, lazy and at risk for child abuse or neglect (Herrman, 2008; Whitehead, 2001). Wiemann et al. (2005) found feeling stigmatised was predicted by being: White race/ethnicity, not engaged or married to the baby's father, socially isolated, aspiring to go to college, verbally abused or ostracised and criticised by family members. Teens who dropped out of school prior to conceiving their child and those who had greater self-esteem were less likely to feel stigmatised.

Although few studies have explored stigma associated with teen fathers, some evidence suggests that young fathers may be evaluated more negatively than teen mothers and with different stereotypes. While teen mothers are depicted as being ‘poor, lone, vulnerable and morally suspect’, teen fathers have been portrayed as ‘being absent, criminal, violent and socially excluded’ (Johansson & Hammarén, 2014, p. 367). Johansson and Hammarén (2014) quoted one teen father who wrote in his blog: ‘You see some raised eyebrows, as a young single father I'm always questioned’ (p. 372). In addition to more negative stereotypes, the role transition to parent may be perceived as more consistent with a young women's developing identity than a young man's who, despite changing social norms, is still expected to assume a primary role as breadwinner even though many young fathers are socio-economically disadvantaged (Berger & Langton, 2011). Negative stereotypes surrounding teen fathers have been shaped by research that has tended to overlook their desire to be good parents and to be involved with their children (Glikman, 2004). Despite common acceptance of stereotypes associated with teen pregnancy and parenthood, recent evidence has suggested that the response of teens to their early role transition is individualistic with many young parents adapting effectively to associated challenges (Weed, Nicholson, & Farris, 2014).

## Measuring social evaluations

Despite the acknowledged importance of social evaluations during the teen years, few studies have investigated attitudes towards pregnant and parenting teens using quantitative measures (for exceptions, see Eshbaugh, 2011; Fulford & Ford-Gilboe, 2004; Kim et al., 2013; Richter, 2013). One study investigated stereotyped perceptions and positivity towards teen mothers with the 21-item Positivity Toward Teen Mothers (PTTM) scale (Eshbaugh, 2011). Stereotyped perceptions were evidenced by endorsement of items reflecting the tax cost associated with teen motherhood, irresponsibility, ignorance and lack of work success. In contrast, the majority of respondents also believed that teen mothers could be good parents and were not neglectful (Eshbaugh, 2011). Predictors of more positive, and less stereotyped, perceptions included not having a teen mother in the family, and class rank; college men and women did not differ in their perceptions of teen mothers.

Further research on the PTTM reduced the scale to 19 items and identified two factors, labelled as Non-judgemental and Supportive (PTTM-Revised; Kim et al., 2013). The nursing students who responded to the survey held positive perceptions about the potential of teen mothers to be good parents, but were more sceptical about the associated taxpayer cost and the degree of responsibility exhibited by teen mothers (Kim et al., 2013). In contrast to findings of Eshbaugh (2011), having a teen mother in the family was related to more positive ratings on the Supportive factor. Although the PTTM (Eshbaugh, 2011) and PTTM-Revised (Kim et al., 2013) have provided an important initial step in accessing social evaluations of teen mothers, this measure is restricted to the cognitive component comprising attitudes and only applies to teen mothers.

The primary objective of the current study was to explore how feelings towards pregnant teens, teen mothers and teen fathers are related to general attitudes and specific beliefs about characteristics and outcomes. This objective represents an initial step in addressing the gap in prior research by expanding our understanding of stereotypes of teen pregnancy beyond parenting teen mothers and by examining both the cognitive and affective components of attitudes. The specific goals of the current study were (1) to assess both cognitive and affective aspects comprising social evaluations of pregnant and parenting teens, (2) to compare the magnitude of negative social evaluations towards teen mothers while pregnant and after birth, with social evaluations towards teen fathers, and (3) to investigate individual differences in social evaluation of pregnant and parenting teens based on sex, race, class rank and prior contact with a teen parent.

## Method

### Participants

The sample included 255 college students between the ages of 18 and 29. To increase the homogeneity of the sample, only data from unmarried students who reported never being pregnant, or raising children, were included. Participants were predominately freshman (42.3%), although 20.2% were sophomores, 22.9% were juniors and 14.6% were seniors. The majority were women (76%). The racial composition of the sample included 63.1% White, 24.3% Black, 5.5% Latino and 7.1% of mixed race or other. Although none of the sample was married, 10.6% reported living with a significant other. Participants were recruited from two public universities in the south-eastern USA. Slightly more than one-half (56.9%) completed the survey online and 43.1% were administered paper copies of survey instruments in small groups. All research procedures were conducted in compliance with APA ethical guidelines.

### Measures

Demographic questions provided information about sex, race and class rank (from freshman to senior). In addition, participants were asked to indicate (1) the youngest age they believed acceptable for men and women to begin childbearing, (2) whether or not they had a close friend or family member who had a teen pregnancy or was a teen parent, and if so, whom, (3) how much they knew about teen pregnancy and/or teen parenting, and (4) what was the source of their knowledge. Participants' cognitive evaluation was measured with the PTTM scale (Eshbaugh, 2011) and a set of semantic differential scales developed specifically for this study. The affective component of their attitudes was measured using feeling thermometers that gauged overall emotional reactions to pregnant teens, teen mothers and teen fathers.

*PTTM* (Eshbaugh, 2011; Kim et al., 2013). Attitudes towards teen mothers were measured by 21 items that reflected both positive (e.g. ‘teen mothers can be good parents’) and negative (e.g. ‘most teen mothers are lazy’) attitudes. All items were rated on a scale from 1 = *strongly disagree* to 4 = *strongly agree*. The development of the scale showed good divergent validity and respectable internal consistency (Cronbach's α = 0.86; Eshbaugh, 2011).

Although previous use of this questionnaire reverse scored negative items to constitute a *non*-judgemental scale, since the majority of these items targeted negative stereotypes, for the current study these 14 items were scored as written with higher ratings reflecting more negative attitudes (Cronbach's α = 0.85). Of the remaining seven items, six were averaged to comprise the Supportive subscale (Cronbach's α = 0.75). The item ‘I believe that most teen mothers should give up their children for adoption’ was included in the Supportive subscale by Kim et al. (2013, p. 988) but was not reliably related to other supportive items within the current sample and was therefore excluded.

*Feeling thermometers*. The affective component of attitudes towards pregnant teens, teen mothers and teen fathers was measured using feeling thermometers. Respondents were asked to think about their general, overall feelings about pregnant teens, teen mothers and teen fathers, and then to describe these feelings on three separate feeling thermometers. Anchors varied from 0 degrees representing *very cold or unfavourable feelings* to 100 degrees representing *very warm or favourable feelings*. Further instructions explained that ratings between 50 and 100 indicated rather favourable and warm feelings, while ratings less than 50 indicated that ‘you don't care too much for this type of person’. Mid-ratings of 50 degrees were associated with feelings that were not particularly warm or cold.

*Semantic differential scales*. Stereotypes about pregnant and parenting teens were assessed using a set of semantic differential scales developed for this study. Each scale included 16 bipolar adjectives (e.g. clueless/competent; immature/mature) separated by a seven-point Likert-type rating scale, and respondents were instructed to rank a representative member of the target group in proximity to one pole or the other. All participants indicated how well the same 12 target adjectives pairs and 4 filler adjective pairs described each of five types of people: adult mothers, adult fathers, teen mothers, teen fathers and pregnant teens. A subsample (*n* = 144) also related the bipolar adjectives to a hypothetical *good parent* and *bad parent*. These good and bad parent ratings provided anchors that were used as a basis of comparison (i.e. how different is a teen mom from an idealised good parent). The ordering of type of person was counterbalanced.

Adjectives were initially chosen based on review of prior research (Breheny & Stephens, 2007a, 2007b; Eshbaugh, 2011; Kim et al., 2013; SmithBattle, 2013). For example, the pair responsible/irresponsible was derived from the PTTM, as was the pair nurturing/neglectful. Adjective pairs were further revised based on a small pilot study of undergraduates. Table 1 lists the 16 bipolar adjectives used in the current study. Four adjective pairs were associated with parenthood, four with maturity or immaturity and four with behaviour problems. In addition, four filler pairs were included as control items (e.g. boring/fun). One-half of the adjective pairs were presented with the positive trait as the higher pole, while the other half had the negative trait as the higher pole. Ratings were recoded for scoring so that higher ratings reflected more positive attitudes. Internal consistency reliabilities (Cronbach's α) of ratings on the 12 targeted adjective pairs were 0.93 for teen mothers, 0.93 for adult mothers, 0.95 for adult fathers, 0.94 for teen fathers, 0.88 for pregnant teens, 0.96 for good parents and 0.97 for bad parents.

**Table 1:** Semantic differential adjective pairs.

	Positive pole	Negative pole
Parenthood	Involved	Uninvolved
	Nurturing	Neglectful
	Warm	Cold
	Loving	Hostile
Maturity	Responsible	Irresponsible
	Sensible	Foolish
	Mature	Immature
	Competent	Clueless
Behaviour	Faithful	Promiscuous
	Moral	Immoral
	Respectful	Rude
	Law abiding	Delinquent
Filler	Physically fit	Out of shape
	Fun	Boring
	Attractive	Plain
	Active	Inactive

## Results

Descriptive information including mean ratings on the PTTM, feelings thermometers and semantic differential scales provided evidence of the positive and negative social evaluations of university students towards pregnant and parenting teens. In addition, correlational analyses explored relationships between affective and cognitive attitudes towards pregnant teens, teen mothers and teen fathers. Expected differences in the direction and magnitude of stereotypes associated with pregnant teens, teen mothers and teen fathers were tested with repeated measures ANOVA. The within-subjects factor was the type of person (i.e. pregnant teen, teen mother and teen father) and the dependent variables were feelings as assessed by feeling thermometers and stereotypes as assessed by the semantic differential scales. Distributions of the repeated variables were examined for deviations of assumptions of sphericity with application of Greenhouse–Geisser (G-G) corrections as needed.

Additional repeated measures ANOVAs were conducted including sex, race, class rank and contact with a friend or family member who had experienced a teen pregnancy as between-subjects factors. Significant main effects or interactions were followed up with univariate analyses, paired samples *t*-tests or independent samples *t*-tests, as appropriate. Analyses investigating race included only White and Black respondents, since there were insufficient numbers in other groups to support valid comparisons. Approximately two-thirds of the sample (67.5%, *n* = 172) reported having a close friend or family member who had a teen pregnancy or was a teen parent. Of these, 121 reported that it was a friend who was a teen parent, 21 reported having a mother or father who was a teen parent, 18 reported a sibling, 56 reported the person was another family member, and 35 reported that the person was an acquaintance or friend of a friend.

### Descriptive analyses

On average, participants reported the youngest acceptable age to begin childbearing was 21.74 years (SD = 2.74) for men and 21.19 years (SD = 2.62) for women. A paired samples *t*-test confirmed that the difference of 0.55 years between young women and men was significant, *t*(243) = 6.41, *p* < 0.001. Furthermore, independent samples *t*-tests indicated that White participants reported significantly lower acceptable ages for women to begin childbearing (*M* = 20.87, SD = 2.43) than Black participants (*M* = 21.93, SD = 3.13; *t*(89.99) = 2.39, *p* = 0.02).

Only 17.9% of the sample reported knowing *a whole lot* about teen pregnancy and/or teen parenting, while 7.6% reported knowing *nothing*. Close to three-fourths of the sample (74.5%) reported knowing *a little bit*. Most participants reported the source of their knowledge to be family life education or child development classes in high school or college (83.4%), from the media (e.g. TV shows about teen mothers; 71%) or from personal experiences (56.6%). Since participants could choose more than one source, numbers do not add up to 100%.

*PTTM.* Scores on the Judgemental and Supportive subscales represent the mean rating of items with a possible range from a low of 1 to a high of 4. Scores above 2.5 reflect general agreement with the items, while those below 2.5 reflect general disagreement. The mean value of judgemental items was 2.48 (SD = 0.39) and the mean value of supportive items was 2.90 (SD = 0.41). Mean values are similar to the 2.71 reported by Eshbaugh (2011), but somewhat lower than the 3.04 derived from information presented for the nursing student sample in Kim et al. (2013). Although distributions for both subscales deviated from normal, skewness and kurtosis indices were all between 1.00 and − 1.00, and all individual scores were within 2 SD of the mean.

As expected, the Judgemental and Supportive subscales were significantly negatively correlated, *r*(253) = − 0.52, *p* < 0.001. Feelings, as measured by the feeling thermometers, were significantly and moderately related to scores on the Judgemental subscale, with somewhat smaller correlations with the Supportive subscale (see Table 2). Stereotyped perceptions, as measured by the semantic differential scales, were also moderately related to scores on both the Judgemental and Supportive subscales.

**Table 2:** Correlations between scores on the PTTM Judgemental and Supportive subscales with feelings and stereotyped perceptions of pregnant and parenting teens (*N* = 255).

Attitude component	Judgemental subscale	Supportive subscale
Feelings towards:		
Pregnant teens	− 0.39^*^^*^	0.25^*^^*^
Teen mothers	− 0.43^*^^*^	0.25^*^^*^
Teen fathers	− 0.41^*^^*^	0.20^*^^*^
Stereotypes of:		
Pregnant teens	− 0.58^*^^*^	0.47^*^^*^
Teen mothers	− 0.48^*^^*^	0.44^*^^*^
Teen fathers	− 0.42^*^^*^	0.19^*^

Note: ^*^^*^ *p* < .01; ^*^^*^^*^ *p* < .001.

Multivariate analyses of variance (MANOVA) with scores on the Judgemental and Supportive subscales as joint dependent variables were used to investigate individual differences in attitudes based on sex, race, class rank and contact. Ratings did not differ significantly by sex, *F*(2, 251) = 0.49, *p* = 0.61, or by class rank, *F*(6, 498) = 1.11, *p* = 0.35. However, a significant multivariate effect was found for race, *F*(2, 220) = 9.18, *p* < 0.001, η^2^_*p*_ = 0.08. Follow-up univariate analyses revealed that ratings by Black participants were significantly higher on the Supportive subscale than ratings by White participants (*M* = 3.11, SD = 0.35 vs. *M* = 2.86, SD = 0.40, *F*(1, 221) = 17.87, *p* < 0.001) but did not differ significantly on the Judgemental subscale.

A significant multivariate effect was also found for contact, *F*(2, 252) = 4.50, *p* = 0.01, η^2^_*p*_ = 0.034. Follow-up univariate analyses indicated that ratings on the Supportive subscale were higher for participants who indicated that they had a friend or family member who had experienced a teen pregnancy (*M* = 2.95, SD = 0.41) compared to those who had not (*M* = 2.79, SD = 0.38). Ratings on the Judgemental subscale did not differ between groups based on contact with a friend or family who had experienced teen parenthood.

Since Black participants were also more likely than White participants to have close contact with teen parenthood through a friend or family member, χ^2^(1) = 15.52, *p* < 0.001, MANOVA analyses comparing judgemental and supportive attitudes based on contact were conducted separately for Black and White participants. Differences in ratings between White participants based on contact with pregnant or parenting friends or family were not significant, but Black participants who had close contact with someone who had a teen pregnancy were both less judgemental (*M* = 2.35, SD = 0.33 vs. *M* = 2.64, SD = 0.30) and more supportive (*M* = 3.14, SD = 0.34 vs. *M* = 2.86, SD = 0.33) than Black participants who had not had contact, *F*(2, 59) = 3.41, *p* = 0.04.

### Comparative analyses of pregnant teens, teen mothers and teen fathers

Overall, participants reported somewhat negative feelings towards pregnant teens (*M* = 39.53, SD = 19.03), teen mothers (*M* = 44.63, SD = 18.82) and teen fathers (*M* = 38.24, SD = 19.71). Repeated measures ANOVA, with G-G correction, revealed that feelings towards teen mothers were significantly more positive than feelings towards pregnant teens, *F*(1, 254) = 43.25, *p* < 0.001, η^2^_*p*_ = 0.15, and towards teen fathers, *F*(1, 254) = 55,39, *p* < 0.001, η^2^_*p*_ = 0.18, but feelings towards teen fathers and pregnant teens did not differ significantly, *F*(1, 254) = 1.70, *p* = 0.19. Inclusion of sex, race or class rank as between-subjects factors in the repeated measures ANOVA did not substantively change the pattern of results; none of the main effects or interactions were significant.

**Figure 1:**
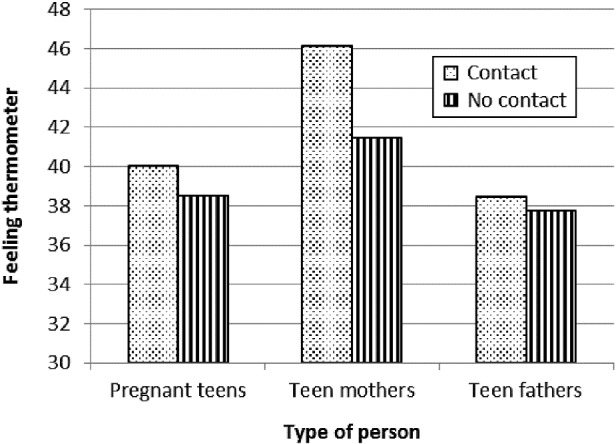
Feeling thermometer ratings of pregnant teens, teen mothers and teen fathers by contact with a friend or family member who had a teen pregnancy or was a teen parent. Ratings below 50 indicate relatively more cold and unfavourable feelings.

Contact with a friend or family member who had experienced teen pregnancy or teen parenthood was marginally related to reported feelings, *F*(1.83, 463.86) = 2.57, *p* = 0.08, η^2^_*p*_ = 0.01. Follow-up independent samples *t*-test analyses indicated that respondents who had contact with a friend or family member with a teen pregnancy felt marginally warmer towards teen mothers (*M* = 46.16, SD = 18.58) compared to respondents who had no contact (*M* = 41.45, SD = 19.04; *t*(253) = 1.88, *p* = 0.06). As shown in Figure 1, ratings of pregnant teens or teen fathers did not differ between groups with and without contact.

**Figure 2:**
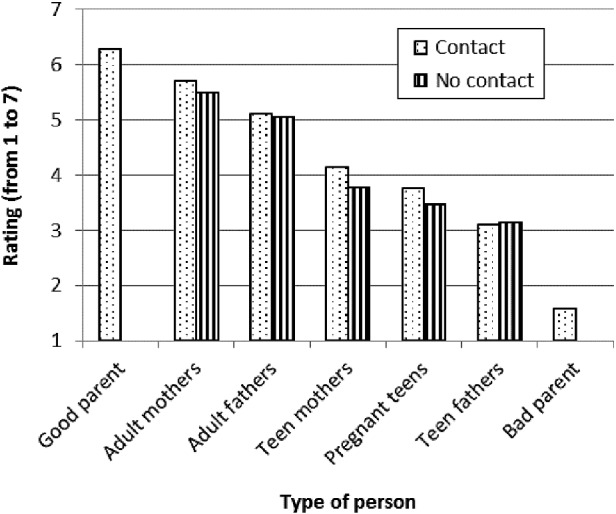
Semantic differential ratings by contact with a friend or family member who had a teen pregnancy or was a teen parent. Higher values indicate positive ratings and lower values indicate negative ratings.

Descriptive analyses of semantic differential ratings, as shown in Figure 2, revealed that adult mothers were rated most favourably (*M* = 5.63, SD = 0.94), followed by adult fathers (*M* = 5.08, SD = 1.17), teen mothers (*M* = 4.03, SD = 1.10), pregnant teens (*M* = 3.66, SD = 0.86), with teen fathers rated most negatively (*M* = 3.12, SD = 1.08). Average ratings between 4 and 7 indicated more favourable than unfavourable responses, and scores between 1 and 4 indicated relatively more unfavourable responses. The average rating of 4 associated with teen mothers indicated significantly less favourable ratings compared to adult mothers, but the rating was not overly negative, and remained considerably higher than ratings associated with the hypothetical bad parent.

Stereotyped perceptions of pregnant and parenting teens were inferred from semantic differential ratings that differed significantly from ratings of adult mothers. Repeated measures ANOVA was used to test the significance and effect size of mean differences in ratings. The main effect for type of person was significant, *F*(1, 254) = 331.55, *p* < 0.001, η^2^_*p*_ = 0.57, using G-G correction. Follow-up contrasts revealed that adult mothers were rated more positively than all other types of people, including adult fathers. Furthermore, pairwise comparisons using Bonferroni correction for the number of contrasts indicated that differences between each type of person were significant (e.g. adult mothers were rated significantly more favourably than adult fathers, and adult fathers were rated significantly more favourably than teen mothers).

Inclusion of gender as a between-subjects factor in the repeated measures ANOVA, with G-G correction, resulted in a small, but significant interaction effect, *F*(2.55, 643.42) = 3.34, *p* = 0.01, η^2^_*p*_ = 0.01. Follow-up independent samples *t*-tests for each type of person revealed that adult mothers were the only type of person rated significantly different between men (*M* = 5.22, SD = 0.96) and women (*M* = 5.77, SD = 0.90, *t*(252) = 4.09, *p* < 0.001. No main effects or interactions with race were found when race was included as a between-subjects factor.

**Figure 3:**
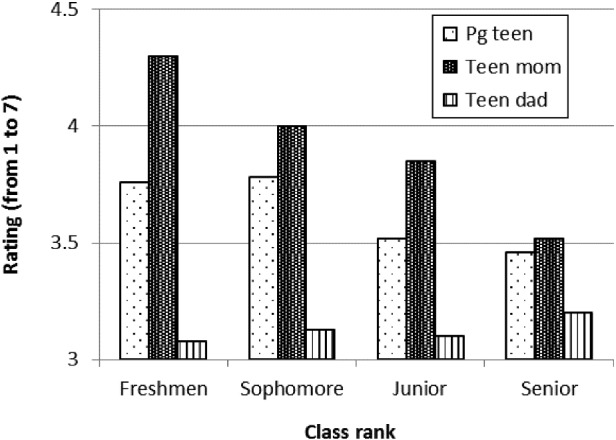
Semantic differential ratings by class rank. Higher values indicate positive ratings and lower values indicate negative ratings.

Inclusion of class rank as a between-subjects factor revealed a significant main effect, *F*(3, 249) = 3.86, *p* = 0.01, η^2^_*p*_ = 0.04, and a marginally significant interaction effect, *F*(7.60, 630.71) = 1.68, *p* = 0.10, η^2^_*p*_ = 0.03. Follow-up pairwise comparisons with Bonferroni correction for multiple comparisons revealed that seniors had more negative stereotypes than freshman (see Figure 3), but that the ratings between other ranks did not differ. Additional univariate analyses, with class rank as the between-subjects factor, were conducted to follow up the marginally significant interaction effect. No significant differences were associated with class rank for pregnant teens, *F*(3, 249) = 2.02, *p* = 0.11, η^2^_*p*_ = 0.02, or for teen fathers, *F*(3, 249) = 0.12, *p* = 0.95, η^2^_*p*_ = 0.00. However, ratings of teen mothers were significantly associated with class rank, *F*(3, 249) = 5.52, *p* < 0.01, η^2^_*p*_ = 0.06. Freshmen rated teen mothers significantly more favourable (*M* = 4.30, SD = 1.05) compared to seniors (*M* = 3.52, SD = 0.98).

Additional analyses of semantic differential ratings that included contact with a friend or family who had experienced a teen pregnancy or teen parenthood as a between-subjects factor revealed a marginally significant interaction effect, *F*(2.46, 543.68) = 2.42, *p* = 0.08, η^2^_*p*_ = 0.01. Follow-up independent samples *t*-test analyses indicated that respondents who had contact with a friend or family member with a teen pregnancy rated pregnant teens, *t*(253) = 2.54, *p* = 0.01, and teen mothers, *t*(253) = 2.50, *p* = 0.01, significantly more favourably compared to respondents who had not had contact, but did not differ on ratings of teen fathers, *t*(253) = − 0.24, *p* = 0.81 (see Figure 2).

## Discussion

Challenges associated with being a teen parent may be exacerbated by the cold and unfavourable feelings endured from others. These negative emotions may be fuelled by beliefs that teen parents possess undesirable traits and characteristics that reflect immaturity, behaviour problems and poor parenting ability (Herrman, 2008; Whitehead, 2001). Although the intention of the stigmatisation may be to deter teen pregnancy (Eshbaugh, 2011), in actuality it may hinder the well-being of young parents. A growing body of research suggests that teens may be particularly sensitive to social evaluation of others and may even integrate other people's perception into their self-concept (Blakemore, 2008; Burnett et al., 2011; Eisenberger et al., 2003; Fulford & Ford-Gilboe, 2004; Masten et al., 2009; Somerville, 2013). In light of the potential consequences of negative stereotypes, the current study contributes to existing literature in understanding both the cognitive and affective components of social evaluation of teen mothers, how attitudes differ towards pregnant teens, teen mothers and teen fathers, and sources of individual differences in social evaluations.

Limiting attitudes towards teen parents to their cognitive component discounts the importance of affect in social evaluation (Esses & Dovidio, 2002; Millar & Tesser, 1992; Ramasubramanian, 2010). Although significant correlations between cognitive and affective aspects of social evaluation, as found in the current study, suggest that these components are moderately related, prior research suggests that beliefs and feelings may have different implications for stigmatising behaviours. Similar to previous research focused on the more cognitive components (Eshbaugh, 2011; Kim et al., 2013), stereotyped perceptions from the semantic differential scale were related to both scales of the PTTM, providing construct validity for both measures. The affective component of social evaluation suggested that those who had colder feelings towards teen parents were likely to be more judgemental and less likely to be supportive.

### Differential social evaluation of pregnant teens, teen mothers and teen fathers

Despite the overall negative perceptions of pregnant and parenting teens, attitudes were differentiated with teen fathers facing the most negative social evaluations. The effect size of 0.57 found for semantic differential ratings suggests that a moderate amount of the variability in positive or negative attributes could be accounted for by the type of person being considered. The negative social evaluation of teen fathers is consistent with the suggestion by Johansson and Hammarén (2014) that teen fathers are farther from the normative ideal of a good parent than are teen mothers. Teen fathers may also be farther from their perceived acceptable age to begin childbearing. Data from both the current sample and more general social norms confirm that men are typically expected to be older than women when they become fathers. For example, men's average age at first childbirth is 25 years compared to 23 years for women in the USA (Martinez, Daniels, & Chandra, 2012), while in Sweden, the average age for men to begin childbearing is 31 years compared to 29 years for women (Johansson & Hammarén, 2014). An alternative perspective for more negative social evaluation of teen fathers is suggested by a recent qualitative study (Weber, 2012). Analyses of reactions of teen fathers to their partners' pregnancies suggested that some young men coped by both denying responsibility and asserting their manhood. While this style of coping may be adaptive in the short term, it also reinforces stereotypes of teen fathers as selfish or even somewhat predatory (Weber, 2012).

In addition, the more negative social evaluation of teen fathers as compared to teen mothers parallels how adult fathers may also be perceived less favourably than adult mothers. Negative ratings for teen fathers may have partially reflected a more general social trend that aligns good parenting with women as opposed to men. A recent survey by the Pew Research Center (Parker & Wang, 2013) found that fewer fathers believed that they were doing an excellent or very good job as a parent compared to mothers (64% vs. 73%). A related survey (‘The New American Father’, 2013) found that the role of providing income was ranked less important for mothers than for fathers; 41% of the respondents indicated that it was an extremely important role for fathers compared to only 25% who indicated that providing income was an extremely important role for mothers. Since younger fathers have had less time to establish a career and are generally less financially secure than older fathers, satisfaction of this role expectation becomes difficult (Berger & Langton, 2011; Glikman, 2004). Belief that young fathers may be unable to provide adequate financial support for their children may be a contributing factor to the more negative stereotypes found for teen fathers and is an important point of future research.

### Individual differences in social evaluation of pregnant and parenting teens

Attitudes towards pregnant and parenting teens were remarkably robust despite individual differences in sex, race, class rank and contact with a friend or family member who had a teen pregnancy or was a teen parent. Feelings towards pregnant and parenting teens did not differ between men and women or between Black and White participants, nor did stereotypes as assessed by semantic differential ratings. Black participants, however, rated supportive items, but not judgemental items, higher compared to White participants. Some research has suggested that teen pregnancy and parenting is more accepted among the Black community and that lower fertility timing norms may be associated with less stigma caused by a teen pregnancy (Geronimus, 2003; Mollborn, 2010). Within the current sample, fertility timing norms, as indexed by the question of the youngest acceptable age to begin childbearing, were significantly lower for White, as compared to Black young adults. This unexpected finding may be limited to the college sample or based on a specific geographic region (i.e. the south-eastern USA).

The contact hypothesis provides an alternative explanation for the more positive support ratings by Black participants (Jackson, 1993) since 88.7% of Black participants reported that a friend or family member had experienced teen pregnancy or parenting as compared to 61.5% of White participants. Prior research has resulted in conflicting conclusions about social evaluations by others with and without contact (Eshbaugh, 2011; Kim et al., 2013). Consistent with findings from Kim et al. (2013) and the contact hypothesis (Jackson, 1993), current results confirm that close contact with a friend or family member who had experienced a teen pregnancy was related to warmer feelings and more positive social evaluation of pregnant teens and teen mothers. Since friends are, by definition, people towards whom we have warm, caring feelings, having friends who were teen parents should be associated with warmer, more favourable feelings.

Class rank was associated with semantic differential ratings, but not feelings towards pregnant and parenting teens. Semantic differential ratings by seniors were more negative than those of freshman and this effect was attributed primarily to ratings of teen mothers. The increasing negativity associated with college progression is inconsistent with results of prior studies utilising the PTTM, although both of these studies reported non-linear associations (Eshbaugh, 2011; Kim et al., 2013). Eshbaugh (2011) reported that evaluations of juniors were most favourable with seniors somewhat less positive, and Kim et al. (2013) reported that seniors were most supportive and less judgemental, while graduate student responses were more negative. All three studies suggest that students become less accepting towards teen mothers as they continue their education.

### Strengths and limitations

The current study extends prior research in several ways. First, in recognition of the affective component of attitudes, feelings towards pregnant and parenting teens were measured. Second, instead of an exclusive focus on teen mothers, the current study differentiated attitudes towards teen mothers from attitudes towards pregnant teens and teen fathers, and included comparisons to adult and good and bad parents. Since participants applied the same set of attributes to both teen and adult parents, the magnitude of the negative stereotype could be quantified. Further research that attempts to modify attitudes will need to consider not just cognitive components of social evaluation, but also affective components.

The homogeneity of the current sample is both a strength and a limitation. The limited age range and relationship status provide an initial understanding of the magnitude of stereotypes by young adults only slightly older than pregnant teens. As two-thirds of the sample reported having a close friend or family member with a teen pregnancy or who was a teen parent, the feelings and beliefs held by university students contribute towards the social evaluation perceived by pregnant and parenting teens. However, it is equally important to assess the attitudes of others who may contribute to the perceived social evaluative threat, including married adults with children and social and health service personnel.

### Implications

Negative social evaluations towards pregnant and parenting teens may impact short- and long-term outcomes in several ways (SmithBattle, 2013). First, evidence from prior research has shown that some pregnant teens delay entry into prenatal care due to shame and embarrassment (Atuyambe et al., 2009; Hueston et al., 2008). Inadequate prenatal care, in turn, is related to increased likelihood of low birth weight, prematurity and other perinatal complications. In contrast, most infants born to pregnant teens who received adequate prenatal care have few complications (Osterman, Martin, Mathews, & Hamilton, 2011). Second, negative social evaluations may make it difficult for pregnant teens to stay in school and finish their education (Luttrell, 2003; Wiemann et al., 2005).

Third, perceived social evaluations may become internalised, affecting both how teens feel about themselves and actual physical functioning (Eisenberger et al., 2003; Masten et al., 2009; Silk, Davis, McMakin, Dahl, & Forbes, 2012; Somerville, 2013). A review by Silk et al. (2012) concluded that perceived social threat, associated with peer rejection, lack of peer support, and social exclusion, was associated with vulnerabilities predisposing youth to anxiety and depression. Evidence from other research confirms that threats of negative social evaluation are linked to long-term physical health problems including rheumatoid arthritis, chronic pain, obesity, diabetes and cardiovascular disease (Murphy, Slavich, Rohleder, & Miller, 2013; Slavich & Irwin, 2014). Pregnant and parenting teens' attunement to social evaluation, therefore, may partially account for links between teen parenting and health outcomes.

Reducing negative social evaluations of pregnant and parenting teens has important implications for society. Continued endorsement of unfavourable attitudes and feelings may actually contribute to less optimal outcomes for teen parents and their children. In contrast, minimising negative social evaluations has the potential to improve prenatal care and subsequent birth outcomes, to enhance educational outcomes, to support positive self-concepts of teen mothers and to improve the mental and physical well-being of teen parents (Atuyambe et al., 2009; Lewis, Scarborough, Rose, & Quirin, 2007; SmithBattle, 2012, 2013).
